# Improved Heat Dissipation of NR/SBR-Based Tire Tread Compounds via Hybrid Fillers of Multi-Walled Carbon Nanotube and Carbon Black

**DOI:** 10.3390/polym15234503

**Published:** 2023-11-23

**Authors:** Mehmet Kodal, Nazlı Yazıcı Çakır, Rumeysa Yıldırım, Nursel Karakaya, Güralp Özkoç

**Affiliations:** 1 Department of Chemical Engineering, Kocaeli University, 41001 Kocaeli, Türkiye; nazliyazici93@gmail.com; 2 Polymer Science and Technology Graduate Program, Kocaeli University, 41001 Kocaeli, Türkiye; yildirimmrumeysa@gmail.com; 3 Sabancı University Nanotechnology Research and Application Center, 34956 Istanbul, Türkiye; guralp.ozkoc@istinye.edu.tr; 4 Xplore Instruments B.V., 6135 KT Sittard, The Netherlands; nursel.karakaya@xplore-together.com; 5 Department of Chemistry, Istinye University, 34396 Istanbul, Türkiye

**Keywords:** NR, SBR, hybrid fillers, distribution, mechanical properties

## Abstract

The development of thermally conductive rubber nanocomposites for heat management poses a formidable challenge in numerous applications, notably within the realm of tire technology. Notably, rubber materials are characterized by their inherently low thermal conductivity. Consequently, it becomes imperative to incorporate diverse conductive fillers to mitigate the propensity for heat build-up. Multi-walled carbon nanotubes (MWCNTs), as reinforcement agents within the tire tread compounds, have gained considerable attention owing to their extraordinary attributes. The attainment of high-performance rubber nanocomposites hinges significantly on the uniform distribution of MWCNT. This study presents the influence of MWCNTs on the performance of carbon black (CB)-reinforced natural rubber (NR)/styrene butadiene rubber (SBR) tire compounds prepared via high shear melt mixing. Morphological analysis showed a good distribution of MWCNTs in the NR/SBR/CB compound. The vulcanization parameters, such as the maximum and minimum torque, cross-linking density, hardness, abrasion resistance, tensile strength, and Young modulus, exhibited a progressive improvement with the addition of MWCNT. Remarkably, adding MWCNT into CB improved the heat conductivity of the NR/SBR/CB compounds, hence decreasing the heat build-up. A percolation mode was also proposed for the hybrid carbon fillers based on the data obtained.

## 1. Introduction

Natural rubber (NR) stands as an exceptionally sustainable material, distinguishing itself as the sole agricultural product within the spectrum of rubber varieties. NR exhibits a myriad of distinctive physical properties, encompassing remarkable attributes like high elasticity, minimal heat generation, exceptional resistance to fatigue crack propagation, and more. These inherent properties render its applicability across a diverse array of engineering domains, including but not limited to its utilization in the construction of tires and shock absorbers [1,2,3]. NR can be effectively compounded with synthetic rubbers to enhance its mechanical characteristics, including tensile strength, resilience, tear strength, fatigue resistance, and fracture toughness. It has been documented that the incorporation of NR into styrene butadiene rubber (SBR) blends can lead to enhancements in oxidative stability [4]. Rubber blends find their most significant application within the tire manufacturing industry. SBR exhibits superior attributes in terms of crack resistance, wet traction, and resistance to adverse weather conditions when compared to natural rubber. Conversely, NR surpasses SBR in terms of strength, heat dissipation, and low-temperature performance. Therefore, the synergistic qualities of NR and SBR have led to their extensive use in the production of tires [5]. It is worth noting that tire compositions typically incorporate substantial quantities of carbon black (CB) to enhance reinforcement and provide desirable properties related to fatigue resistance and abrasion [6]. Nevertheless, the utilization of these fillers poses certain challenges. Their relatively high density contributes to an increase in the specific gravity of rubber compounds. Furthermore, their efficacy in reinforcing rubber materials is most pronounced when employed at substantial loadings [7,8,9]. It is essential to note that, in the context of NR vulcanizates, an increase in CB ratios is accompanied by considerable heat build-up, which, in turn, adversely affects various performance attributes of vehicle tires. Enhancing the performance of vehicle tires entails addressing issues such as the mitigation of heat accumulation, the augmentation of wet handling characteristics, and the reduction in rolling resistance [10,11]. In pursuit of these objectives, researchers have explored the application of hybrid nanofillers, encompassing the use of CB or combinations of two or three distinct fillers. This approach has garnered attention for its potential in the development of advanced, high-performance rubber composites [6,12,13,14]. Researchers have undertaken investigations into the partial substitution of CB with carbon nanotubes (CNTs). Their studies have unveiled a synergistic interaction between CNT and CB, resulting in a significant enhancement in crack propagation resistance within NR composites. Moreover, this concurrent usage of CNT and CB has demonstrated notable efficiency in mitigating heat build-up in NR vulcanizates [2,15].

CNTs are cylindrical molecular structures composed of carbon, characterized by nanometer-scale diameters and micrometer-scale lengths. Their extraordinary mechanical properties, such as a remarkably high aspect ratio, expansive surface areas, exceptional tensile strength of 150–180 GPa, moduli ranging from 640 GPa to 1 TPa, and remarkable elasticity, endow them with substantial reinforcing capabilities [7,9,16,17,18]. The enhancement of properties in polymer/CNT composites primarily hinges on factors including the uniform dispersion of CNTs, interactions between the filler and the matrix, and the inherent attributes of the CNTs themselves. Due to relatively weak intermolecular forces such as van der Waals and π-π interactions that govern the assembly of CNTs, these nanotubes exhibit a propensity to intertwine, forming sizeable aggregations referred to as “bundles” [19,20,21]. The attainment of high-performance NR nanocomposites characterized by homogeneous dispersion of nanofillers necessitates the resolution of two prominent challenges: aggregation and inadequate matrix–filler interactions. The establishment of robust interactions between NR and nanofillers, along with the effective alleviation of filler–filler agglomerations, is of paramount importance in realizing property enhancements within NR-based nanocomposites. Several well-regarded processing techniques for the fabrication of NR nanocomposites include methods such as melt/mechanical mixing, solution mixing, and latex casting [22]. 

Within the solution mixing method, the polymer solution is intricately combined with the nanofiller dispersion through means such as ultrasonication, stirring, or shear mixing. Subsequently, the resultant polymer–nanofiller dispersion is cast into a mold and allowed to undergo solvent evaporation. This approach offers the advantage of achieving a more refined nanofiller dispersion. Nevertheless, the removal of solvents upon completion of this procedure presents a notable challenge. Moreover, the method is afflicted by drawbacks such as elevated costs, substantial solvent consumption, and environmental concerns related to solvent disposal [22,23]. Fakhrúl-Razi et al. [24] undertook the preparation of nanocomposites comprising NR and multi-walled carbon nanotubes (MWCNTs) through the solution casting method. Toluene was used as the solvent for dispersing the MWCNTs, and NR was similarly dispersed in toluene. The two solutions were subsequently amalgamated, resulting in a final solution containing a well-balanced blend of MWCNTs within the NR matrix, which underwent further processing. This methodology facilitated the attainment of a uniformly dispersed MWCNT structure within the NR, thereby yielding enhancements in mechanical, chemical, and physical properties. In another study, the fabrication of NR/MWCNT nanocomposites was executed through the incorporation of MWCNTs at various loading levels, employing two distinct methodologies: mechanical mixing and solution mixing by Hanafi Ismail et al. [25] The process entailed the initial swelling of natural rubber in toluene under sustained agitation. Concurrently, multi-walled carbon nanotubes were dispersed in toluene and subjected to magnetic stirring for a duration of 20 min. Following this interval, the MWCNT dispersion was amalgamated with the rubber–toluene solution and agitated vigorously for a period of 2 h. Subsequently, the solvents within the resultant dispersions were subjected to evaporation within a fume chamber until the weight of the rubber-MWCNT composite sample reached a constant value. Once this criterion was met, the sample was removed from the chamber and prepared for further mixing with additional rubber additives, accomplished on a laboratory-scale two-roll mill, resulting in an improved MWCNT dispersion in the rubber matrix [25]. In an effort to enhance the dispersion of MWCNTs within the NR matrix, a solution-mixing method was undertaken by Huang et al. [26]. Initially, MWCNTs were exfoliated and dispersed in water through ultrasonication, with the aid of tannic acid (TA) serving as a dispersing agent. Subsequently, this MWCNT dispersion was combined with interspersed SiO_2_ microspheres (m-SiO_2_). It is noteworthy that a notable attribute of this process is the ability to uniformly disperse both m-SiO_2_ and MWCNTs within the NR matrix through direct mixing of the dry hybrid filler and NR within a double roller mill. This method capitalizes on the distinctive and synergistic dispersing effects between m-SiO_2_ and MWCNTs, in addition to the dispersing efficacy conferred by TA. The ultimate research findings underscore the excellent mechanical, electrical, and thermal properties [26].

The latex mixing method involves the utilization of rubber in its latex form. The dispersion of nanofillers is integrated with the polymer latex and subsequently undergoes a casting and drying procedure. Alternatively, latex coagulation can be employed to foster substantial interactions between the nanofiller and the polymer matrix. In this particular context, the coagulation of the latex and the nanofiller dispersion is achieved through the addition of an acidic agent, followed by the incorporation of additional curatives into the polymer nanocomposite utilizing mechanical techniques. It is noteworthy that this method obviates the necessity for highly toxic and expensive organic solvents, thereby establishing itself as an ecologically sustainable alternative to solution mixing, as elucidated [22,27]. Nonetheless, the substantial volume of deionized water employed for the dispersion of CNT results in the dilution of the latex, inducing a propensity for CNTs to settle within the diluted latex medium and, consequently, giving rise to a secondary agglomeration phenomenon [28,29]. The preparation of nanocomposites based on NR has been accomplished through a latex stage mixing technique featuring the incorporation of single-walled carbon nanotubes (SWNTs) by Anoop Anand K. SWNTs were initially dispersed in water using ultrasonication and subsequently stabilized through the introduction of a surfactant. This stabilized aqueous dispersion of SWNTs was then blended with NR latex to investigate the impact of the nanofiller on the rheological properties of the NR latex. Subsequent to the compounding of the latex and the curing process, high-quality composite films were obtained, characterized by a pronounced improvement in their mechanical properties. Remarkably, the composite film, including 2 phr SWNTs, exhibited a substantial increase of 56% in tensile strength and a 63% increase in modulus in comparison to pure NR films [30]. In another study, Laiyun Wei et al. stated that the utilization of the latex mixing method was pivotal in enhancing the dispersion of graphene oxide (GO)/CNT hybrid fillers and CB within the NR matrix. This enhanced dispersion mechanism contributed to the development of a more robust and efficient filler network, consequently leading to significant enhancements in the modulus and energy dissipation properties of NR composites. The introduction of GO/CNT hybrid fillers and CB into the NR matrix resulted in a substantial improvement in resistance to fatigue crack propagation and a reduction in heat generation, thereby enhancing the overall performance of NR composite materials [2]. In another work, polyvinylpyrrolidone (PVP) was employed as a dispersant for MWCNTs, and the effectiveness of various dispersion techniques, including high-speed shear, water bath ultrasonication, and tip ultrasonication, was systematically compared to assess their exfoliation capabilities. Following this, the appropriateness of employing nonionic PVP for the purpose of achieving high-speed shear exfoliation and dispersion of MWCNTs was thoroughly investigated and fine-tuned, while classical anionic sodium dodecyl benzene sulphonate (SDBS) and cationic cetyltrimethylammonium bromide (CTAB) were utilized as control agents for comparative assessment. Ultimately, an aqueous dispersion of MWCNTs, which had been noncovalently modified by PVP, hereinafter denoted as PVP-m-MWCNTs, was amalgamated with natural latex to produce a composite material comprising NR and MWCNTs, designated as W-NMC. A comparative assessment, conducted in relation to the conventional dry-mixing procedure (D-NMNCs), elucidated that the wet mixing methodology implemented in this investigation yielded W-NMCs distinguished by their superior tensile properties, reduced compression temperature rise, enhanced resistance to DIN abrasion, increased electrical and thermal conductivity, augmented resilience against ice and wet-slip, and diminished rolling resistance. These improvements can be attributed to the enhanced dispersion of MWCNTs and the reinforcement of the interfacial interaction between NR-MWCNTs within the W-NMNCs, aligning with the research findings of Wei Xiao [31]. An investigation conducted by Song et al. focused on the development of high-performance nanocomposites comprising MWCNTs and SBR through a latex mixing process, with the utilization of lower MWCNT quantities. To augment MWCNT dispersion in the aqueous phase for latex mixing, a diverse array of surfactants was systematically employed to modify the MWCNTs. In a targeted approach, a suspension of MWCNTs was incorporated into the SBR latex to optimize the dispersion of MWCNTs within the elastomeric matrix, consequently bolstering interfacial strength. The successful realization of uniformly dispersed MWCNTs throughout the SBR matrix yielded composite materials distinguished by their exceptional mechanical, thermal, and electrical properties, even at low MWCNT loadings [32].

In the melt/mechanical mixing method, the production of nanocomposites is achieved through the utilization of equipment such as an internal mixer, two-roll mill, ball mill, or similar apparatus. This method finds substantial favor within industrial applications owing to its environmentally conscientious attributes, cost effectiveness, elevated efficiency, and expeditious production capabilities. Notably, the solvent-free characteristic represents a pivotal advantage, rendering it a highly regarded alternative to solution mixing [22,23]. Hanafi Ismail et al. prepared NR/MWCNT samples by using a laboratory-sized two-roll mill in accordance with ASTM D3184 guidelines [33]. The authors found a more favorable dispersion of MWCNTs within the NR matrix when employing the solution mixing method, in contrast to the mechanical mixing method. In terms of mechanical properties, the tensile modulus exhibited a direct proportionality with the MWCNT loading, while conversely, the tensile strength and elongation at break displayed an inverse relationship. Importantly, NR/MWCNT nanocomposites prepared via the solution mixing method exhibited superior tensile characteristics in comparison to composites produced through mechanical mixing techniques [25].

Laboratory-scale formulation development traditionally relies on small-scale internal mixers and two-roll mills, which are encumbered with several significant drawbacks. These conventional compounding methods entail labor-intensive procedures, requiring at least two personnel for the preparation of a single batch. Furthermore, these methods pose problems in terms of contamination, especially when dealing with different types of samples, necessitating separate Banbury machines for black and nonblack samples. Moreover, there is an extended turnaround time involved in the process. In addition to these operational challenges, safety concerns are associated with the open mixing area of two-roll mills. This process typically demands a substantial material volume, thus exacerbating the cost implications, especially when dealing with expensive additives. Moreover, the spatial requirements of both open mills and internal mixers are substantial, consuming approximately 6 square meters of laboratory space, particularly due to the dusting associated with carbon black additives. However, the adoption of high-torque laboratory twin-screw micro-compounders, which have served the plastic industry for over three decades, presents a more efficient alternative for the formulation of new rubber compounds. This method streamlines sample preparation, contributing to the overall cost effectiveness of research and development efforts. Micro-compounders offer the advantage of being operated by single personnel, characterized by ease of operation, reduced labor intensity, and a remarkably expedited compound preparation time, typically as short as 5 min. These devices require only minimal quantities of materials, which is particularly advantageous when working with costly additives. Additionally, micro-compounders enable the formulation of more precise compound compositions and occupy significantly less laboratory space compared to their conventional counterparts. Their ease of cleaning and enhanced safety features further underscore the advantages of this modern approach to rubber compounding at the laboratory scale.

Hence, the primary aim of this research is to investigate the influence of a hybrid microstructure developed within a tire tread compound composed of an NR/SBR blend and augmented by the inclusion of MWCNT and CB. This study seeks to elucidate the impact of this hybrid filler system on various properties, encompassing the mechanical, thermal, rheological, morphological, and electrical and thermal conductivity aspects of the composites. The distribution of these hybrid fillers within the NR/SBR matrix was accomplished through the use of a high-torque lab-scale twin-screw micro-compounder, a method until now unreported in the literature. Within this context, MWCNTs were introduced into the NR/SBR composite, in conjunction with the reinforcing CB, within the formulation of a tire tread composition. This approach aims to provide deeper insights into the intricate dynamics of this composite system, particularly with regard to the structure-property relationships arising from the distinctive synergism inherent in a macro-nano dual filler system. 

## 2. Materials and Methods

### 2.1. Materials

NR and SBR were purchased from Baria Rubber, Ho Chi Minh City, Vietnam. N330-type CB was purchased from Elkim, Istanbul, Türkiye. Insoluble sulfur, zinc oxide, stearic acid, N-(cyclohexylthio)phthalimide (PVI), 2-mercaptobenzothiazole (MBT), and N-cyclohexyl-2-benzothiazolesulfenamide (CBS) were used for the vulcanization system. 2,2,4-trimethyl-1,2-dihydroquinoline (TMQ) and N-Isopropyl-N′-phenyl-1,4-phenylenediamine (IPPD) were used as antiaging agents. Naphthenic oil was obtained from Petro Yağ, Kocaeli, Türkiye. MWCNT, with an average diameter of 8–10 nm, a length of 1.5 μm, a purity of 92%, and an electrical conductivity of 98 S/m, was purchased from Nanografi, Ankara, Türkiye (Table 1). According to the manufacturer of MWCNT used in this study, it was obtained via the chemical vapor deposition method. 

### 2.2. Processing

**Figure 1:**
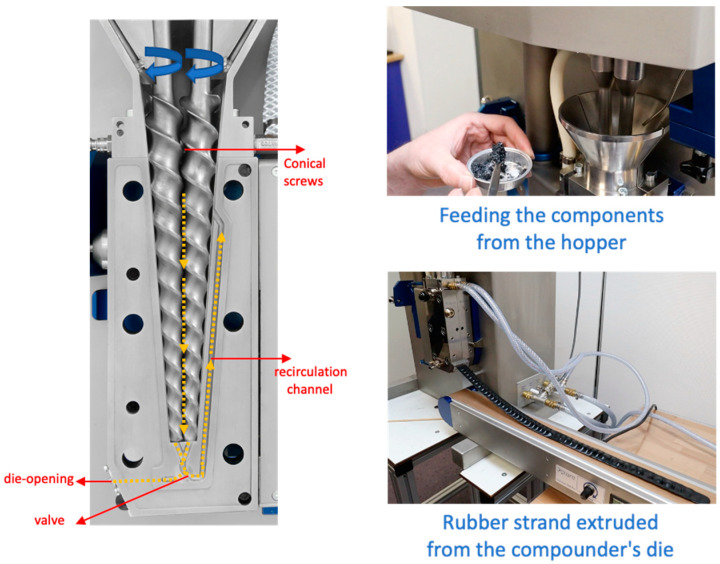
MC 15 HT twin-screw micro-compounder (Xplore Instruments, The Netherlands).

A laboratory-scale high-torque twin-screw micro-compounder, namely, an Xplore Instruments MC 15 HT (from Sittard, The Netherlands), was employed for the preparation of the formulations, namely, the NR/SBR-based compounds. Initially, natural rubber (NR) was subjected to mastication within the MC 15 HT for a duration of 30 s, with the addition of peptizer as a processing aid. Subsequently, the premasticated NR was combined with styrene butadiene rubber (SBR), carbon black (CB), multi-walled carbon nanotubes (MWCNT), and other constituents, excluding sulfur and accelerators. This compound was prepared within the MC 15 HT micro-compounder, operating at a rotational speed of 100 rpm. The temperature of the machine’s barrel was meticulously regulated via water-cooling jackets to maintain it at a constant temperature of 60 °C. Following an elapsed time of 2 min, sulfur and accelerator agents were introduced into the compound, and a mixing phase of 1 min was ensured. The resulting rubber strands were subsequently extricated from the compounder’s die by adjusting the position of the valve. An illustration of the compounding equipment can be observed in Figure 1. The vulcanization process for the NR/SBR composites was executed within a hot press, sustaining a temperature of 160 °C. The optimal curing temperature and duration were ascertained through analyses employing a differential scanning calorimeter (DSC) and a moving die rheometer (MDR), respectively. Recipe 1, Recipe 2, and Recipe 3 correspond to the MWCNTs contents of 0, 3, and 7 phr. The total amount of filler is 30 phr for each sample, as seen in Table 2.

### 2.3. Characterization

The assessment of CB and MWCNT distribution within cured NR/SBR composites was conducted through the utilization of scanning electron microscopy (SEM) and DisperGrader analysis. In the SEM examination, the composite surfaces underwent a gold coating process to prevent sample charging artifacts. Additionally, the assessment of CB and MWCNT distribution within the NR/SBR matrix, as well as the overall distribution quality of the cured NR/SBR samples, was carried out in accordance with the ISO 11345 Method C standard [34], employing DisperGrader analyses. Five tests on different parts of each test piece were carried out for DisperGrader analyses. 

The investigation of the thermal properties of NR/SBR compounds was undertaken using a differential scanning calorimeter (DSC), specifically a Mettler Toledo DSC-1 Star instrument. DSC analyses were executed within a controlled N_2_ atmosphere, employing a gradual heating rate of 10 °C per minute over a temperature range between 25 °C to 300 °C.

Significant rheological parameters such as optimum cure time, scorch time, and cure rate index (CRI) were derived through the analysis of rheometer curves. A moving die rheometer (MDR) from Alpha Technologies was employed for these purposes, following the guidelines outlined in ASTM D-5289 [35]. The rheometer curves, specifically collected at an elevated temperature of 160 °C, were employed to characterize the pertinent rheological parameters. CRI, a measure of the speed of vulcanization reaction, was calculated using the following Equation (1):

(1) $$CRI=\frac{100}{t_{90}-t_{s2}}$$

where $t_{s2}$ is the scorch time and $t_{90}$ is the optimum cure time.

The cross-link density (CLD) of the vulcanizates was subsequently calculated through the application of the Flory–Rehner equation. This computation involved the utilization of key factors such as swelling ratios in toluene, the density of the rubber matrix, volume fractions pertaining to both the polymer and solvent, as well as the critical polymer–solvent interaction parameter, ꭕ. The densities of Recipes from 1 to 3 were measured as 1.052, 1.012, and 0.997 g/cm^3^, respectively. In addition, solvent–polymer interaction value of all Recipes is 0.350. 

The determination of cross-link densities in rubber materials can also be achieved through the examination of stress relaxation behaviors in accordance with the classical rubber elasticity theory based on the neo-Hookean law. According to ASTM D8363-20 [36], crosslink density can be deduced from TSSR-measurements by considering the initial slope of the stress-temperature curve. This method is based on the neo-Hookean material model, which applies well to ideal rubber. The initial slope of the stress–temperature curve, the absolute value of initial stress, $\sigma_{0}$, was used to calculate the crosslink density using Equation (2) given below:

(2) $$\nu =\frac{\sigma_{0}}{RT\left[\lambda -\lambda^{-2}\right]}$$

The thermoelastic behavior of vulcanizates is examined at constant elongation in a temperature range from 30 °C to 120 °C. When a stretched elastomer sample is heated, there is an increase in stress, which, in filled elastomers, is superimposed by physical and chemical relaxation processes and is partially compensated for [37]. The proportion of the polymer layer adsorbed on the filler surface is also considered. It is assumed here that the polymer segments are immobilized within the adsorbed polymer layer at room temperature, which leads to an apparent increase in the filler content. For the experimental execution, a constant tensile strain of 50% was applied to a dumbbell-shaped test specimen (Type 5A, ISO 527 [38]). Subsequently, the specimen underwent a preconditioning phase, allowing for isothermal relaxation at 30 °C over a duration of 2 h without any additional heating. Following this initial stage, the specimen was subjected to a controlled heating process, commencing at 30 °C and concluding at 120 °C, with a heating rate of 3 °C per minute. This process continued until full stress relaxation was achieved. The relaxation spectrum H(T) is calculated from Equation (3), the negative derivative of the non-isothermal relaxation modulus, $E_{non-iso}=\sigma /\varepsilon_{0}$, which, at a constant heating rate, β=∆T/t, follows from the tensile stress ascertained, σ(T).

(3) $$H\left(T\right)=-∆T.\;{\left[\frac{{dE}_{non-iso}}{d∆T}\right]}_{\beta }$$

The tensile properties of the cured composites composed of NR and SBR were determined through the utilization of an Instron Universal Testing Machine (Model 3345), with strict adherence to the standards set forth by ASTM D412 [39]. The testing was conducted at a crosshead speed of 500 mm/min. To gauge the hardness of the samples, measurements were performed using a Zwick Shore A-type durometer, adhering to the specifications outlined in ASTM D2240 [40]. To characterize the abrasion resistance of composites, a DIN Abrasion test was carried out. This assessment followed the guidelines established by ISO 4649 [41] and employed a DIN abrasion resistance tester. The methodology is based on evaluating the volume changes induced by the frictional forces arising from the abrasive action.

Thermal stability of the composites was determined using thermal gravimetric analysis (TGA, Mettler Toledo), under a N_2_ atmosphere and at a heating rate of 10 °C/min from ambient temperature to 600 °C.

The thermal conductivity of the disk-shaped CB and CB/MWCNT-reinforced NR/SBR composites was carried out according to the ASTM E1530 standard [42]. Multiple trials, exceeding three in number, were undertaken for each sample. Surface resistivity measurements were conducted on compression-molded specimens with a 15 V potential difference, and a current of up to 1.5 mA was applied between parallel conductive probes. Measurements were performed at standard room-temperature conditions.

## 3. Results and Discussions

### 3.1. Morphological Analyses of Samples via Scanning Electron Microscopy (SEM) and DisperGrader

**Figure 2:**
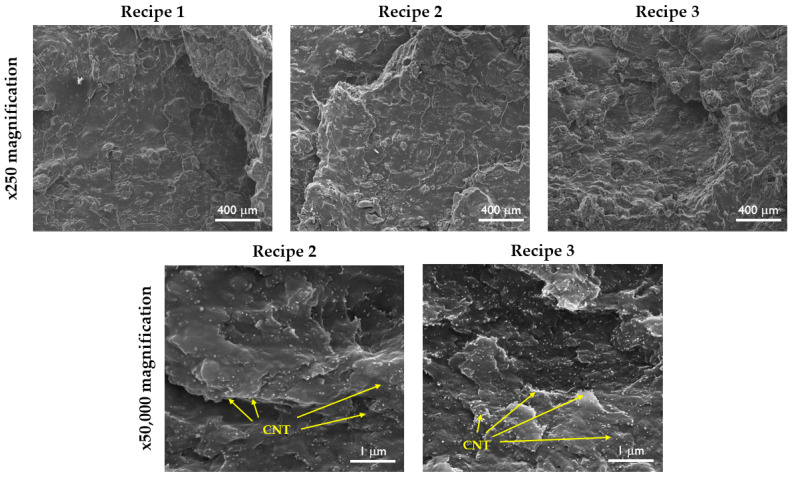
Cryogenically fractured surface morphologies of NR/SBR/CB and NR/SBR/CB/MWCNT composites.

**Figure 3:**
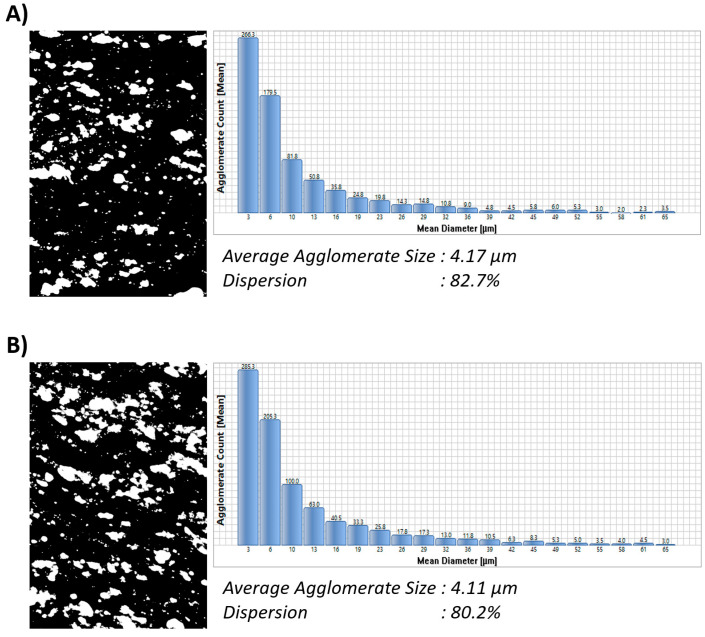

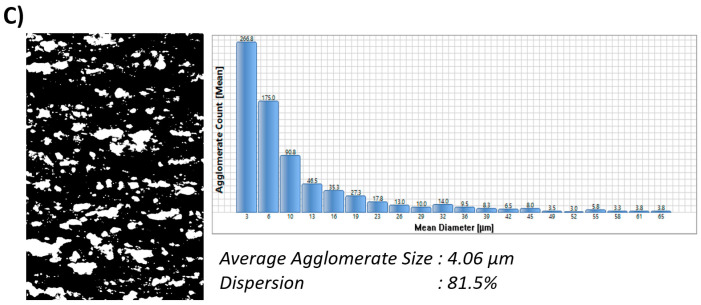
Carbon black dispersion in NR/SBR and NR/SBR/MWCNT composites; (**A**) Recipe 1, (**B**) Recipe 2, (**C**) Recipe 3, (Magnification 100×).

To achieve the desirable properties of CNT-reinforced polymer composites, it is important to establish a homogeneous distribution of CNTs in the polymer matrix. When the distribution of CNTs fails to meet the desired criteria, it impedes the complete realization of their reinforcing potential. As seen in Figure 2, for both CB and CB/MWCNT-reinforced NR/SBR composites, a smooth surface morphology was obtained. In a broader context, the distribution of carbon black within the polymer matrix is intricately linked to the diminishment of carbon black aggregate dimensions. It is evident that the distribution of carbon black is uniform across all compound compositions. It is widely acknowledged that achieving uniform distribution of CNTs within a polymer matrix poses a substantial challenge when employing conventional polymer processing techniques, such as two-roll mixing, Banbury mixing, extrusion, and injection molding. This challenge arises from the intricate entanglement and robust π-π stacking interactions prevalent among CNTs [26,43]. As seen in Figure 2, the SEM images reveal a homogeneous distribution of MWCNTs within the NR/SBR matrix, displaying an absence of discernible aggregation. Furthermore, an examination of the cryogenically fractured surfaces of the NR/SBR/CB/MWCNT composites demonstrates smoother morphology. Moreover, the concentration of MWCNT particles increased as the MWCNT content increased as expected. Furthermore, it is noteworthy that all rubber compounds displayed a nearly identical average particle size. Moreover, the distribution of carbon black was quantitatively assessed via DisperGrader analyses, yielding an 80% distribution, which attests to the commendable homogeneity observed in all composite materials (Figure 3).

The phase structure of the particulate-filled composite materials is subject to influence from several pivotal factors, including surface characteristics, viscosity, the blend ratio of individual components, and the intricacies of the compounding process [44]. The flow characteristics within an intermeshing corotating twin-screw extruder primarily manifest as a state of simple shear in the radial direction, coupled with the presence of elongational flow in the axial direction [45,46]. In contrast to a Banbury mixer, it is noteworthy that the entire composition within an intermeshing twin-screw corotating extruder undergoes continuous exposure to shear stresses throughout the processing cycle. This resulted in a homogeneous distribution of MWCNT and CB within the polymer matrix. Moreover, the synergistic intercalation among string-like CNTs and CB particles serves to effectively mitigate their reagglomeration tendencies, culminating in the establishment of a well-developed network architecture. This phenomenon consequently lends credibility to the assertion that the distribution of carbon black fillers in the NR matrix has been significantly enhanced [2,10,47]. 

### 3.2. Curing and Rheological Properties of Compounds

**Figure 4:**
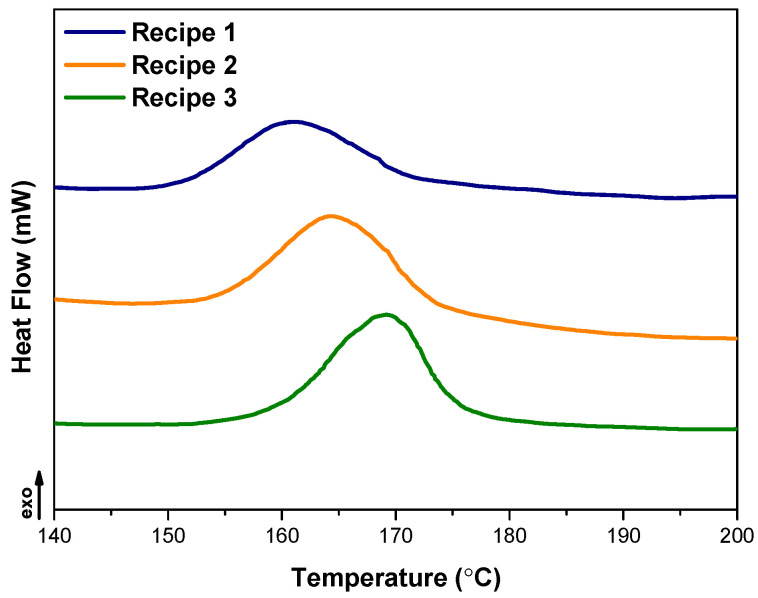
DSC thermograms of compounds.

In order to investigate the influence of CB and CB/MWCNT hybrid fillers on the thermal properties of NR/SBR tire tread compounds, an examination was conducted using differential scanning calorimetry (DSC) on uncured NR/SBR compounds. The DSC curves are graphically presented in Figure 4, and the results are tabulated in Table 3. Evidently, all examined samples demonstrated exothermic peaks attributed to the cross-linking of the materials. Notably, the reaction enthalpy exhibited a proportional increase corresponding to the escalating carbon nanotube content within the compounds. Furthermore, a discernible shift toward higher values was observed in the onset temperature of crosslinking (T_x-link,onset_). This phenomenon can be ascribed to the elevated viscosity and barrier effect induced by CNT nanoparticles, consequently delaying the initiation of the vulcanization reaction [48]. Moreover, several studies have suggested that the incorporation of CNTs may result in the absorption of fundamental accelerator species, leading to a retardation of the vulcanization process [49,50]. In summary, it can be deduced that augmenting the quantity of MWCNTs and achieving a uniform distribution of MWCNTs within the matrix increases the adsorption of accelerator compounds, thereby postponing the vulcanization process.

**Figure 5:**
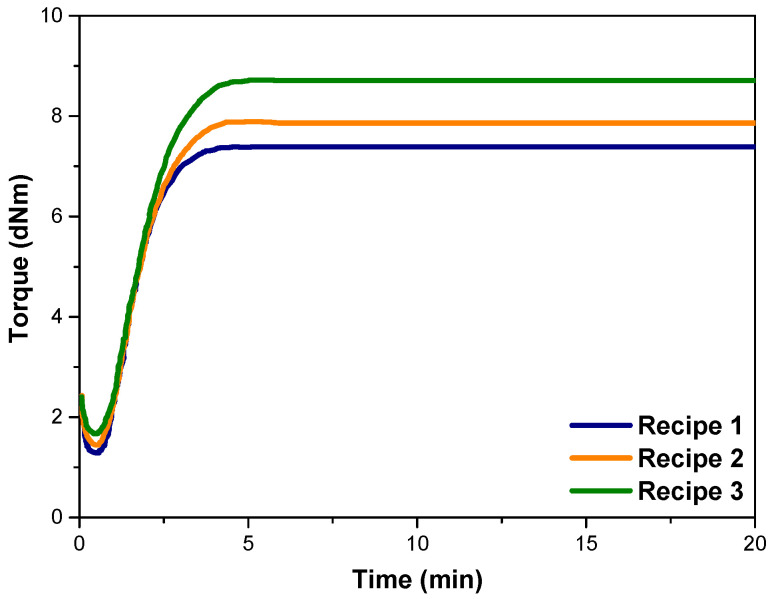
Rheometer curves of compounds.

Rheological assessments were conducted at a selected curing temperature of 160 °C, yielding several key parameters, including the scorch time (t_s2_), optimum cure time (t_90_), cure extent, maximum and minimum torque values (MH and ML), as well as the cure rate index (CRI). The term “cure extent” signifies the cross-link density (CLD) of a vulcanizate and is calculated as the disparity between the MH and ML values. A summary of these significant findings is provided in Table 4. The graphical representation in Figure 5 illustrates the rheometer curves consisting of CB and CB/MWCNT hybrid fillers reinforced NR/SBR composites exhibited the ability to attain a plateau region immediately following the vulcanization process.

The scorch time, denoted as t_s2_, represents the premature vulcanization of rubber samples. As seen in Table 4, the scorch time of NR/SBR vulcanizates exhibited an increase when MWCNT nanoparticles were incorporated into NR/SBR. Notably, this effect is more pronounced with the addition of a higher content of MWCNT. Therefore, it can be concluded that there is a discernible delay in the scorch time, providing a more generous processing window in the presence of MWCNTs. This delay in scorch time can be attributed to the strong adsorption capacity of the CNT surface for vulcanization agents and accelerants, which, in effect, postpones the onset of vulcanization processes [51,52]. Similarly, the optimum cure time prolongs from 2.78 min to 3.00 min and 3.19 min upon the addition of 3 phr and 7 phr of MWCNTs, respectively. This indicates that the addition of MWCNTs prolonged the duration required for the complete curing reaction, which is consistent with lower cure rate index values. This situation can be attributed to the fact that the introduction of well-distributed MWCNT nanoparticles in the NR/SBR matrix permits an adequate timeframe for the development of chemical interactions between MWCNTs and the rubber matrix. This, in turn, facilitates the completion of the vulcanization reaction, leading to the establishment of a robust cross-link network, thereby enabling efficient load transfer between the filler and the rubber matrix [51].

The minimum torque value aligns with the initial viscosity characteristics of the blends comprising unvulcanized rubber and filler. As the concentration of MWCNTs in the blend rises, the minimum torque value (ML) exhibits a discernible increment (Table 4). Throughout the process of curing, the maximum torque (MH) exhibited a progressive rise in the NR/SBR compounds, including MWCNTs. Notably, the time required to attain maximum torque in the nanocomposite containing MWCNTs exceeded that of the NR/SBR/CB. This observation suggests a comparatively slower vulcanization rate for NR/SBR/MWCNT nanocomposites, a trend corroborated by findings from cure rate index values. Concurrently, it is worth noting that the NR/SBR nanocomposites with 7 phr MWCNT nanoparticles demonstrated the highest maximum torque among the materials under consideration. This phenomenon can be attributed to the exceptionally high modulus and higher surface area of MWCNTs, which effectively constrained alterations in the molecular configuration of the polymer and, consequently, increased the modulus of the rubber composites [49,53]. Furthermore, subjecting the composite to elevated torque during the compounding process has enabled the homogeneous distribution of MWCNTs within the rubber matrix, which leads to an increase in the interactions between the rubber and filler components. This, in turn, culminated in an improvement in the reinforcement of the NR/SBR matrix. It is evident that the addition of MWCNTs in NR/SBR/CB compounds promoted the formation of a network structure. This observation aligns with the variations in minimum and maximum torque (cure extent values), which are typically indicative of cross-link density, as shown in Table 4. This was due to the enhanced distribution of MWCNTs in the rubber matrix. As mentioned in the literature, a higher value for MH–ML difference serves as an indicator of superior MWCNT distribution. This is substantiated by the fact that effective cross-linking within the entrapped rubber clusters within MWCNT aggregations can be significantly impeded [31,54,55,56,57,58]. 

Recipe 3 (MWCNT loading of 7 phr) reveals the highest ML (viscosity index), MH (crosslinking density index), and, consequently, difference in torque (strength index) compared to those with a lower content of MWCNT. This observation has confirmed the possible diffusion of curatives from the SBR phase to the NR phase with higher crosslinking density. 

### 3.3. Cross-Link Density Measurements and Stress Relaxation Behaviors of Samples

**Figure 6:**
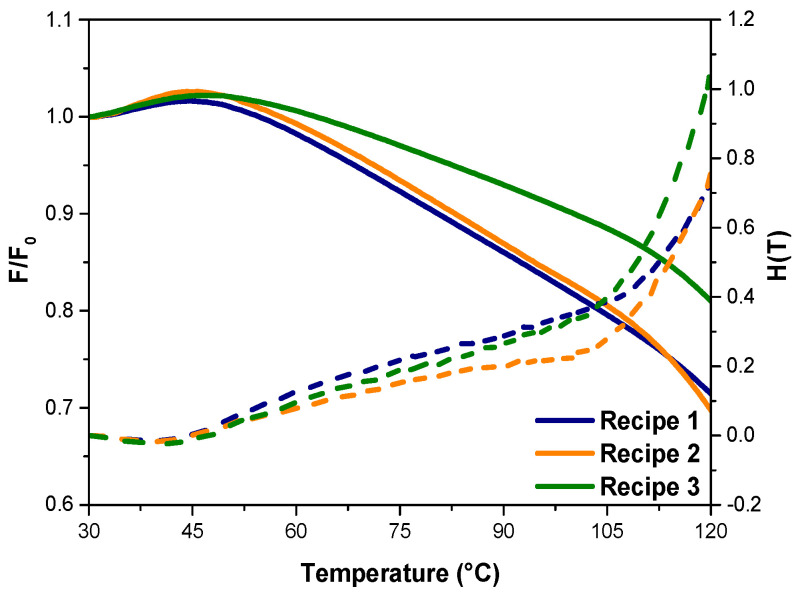
Stress–temperature curves at a constant strain for NR/SBR compounds.

**Figure 7:**
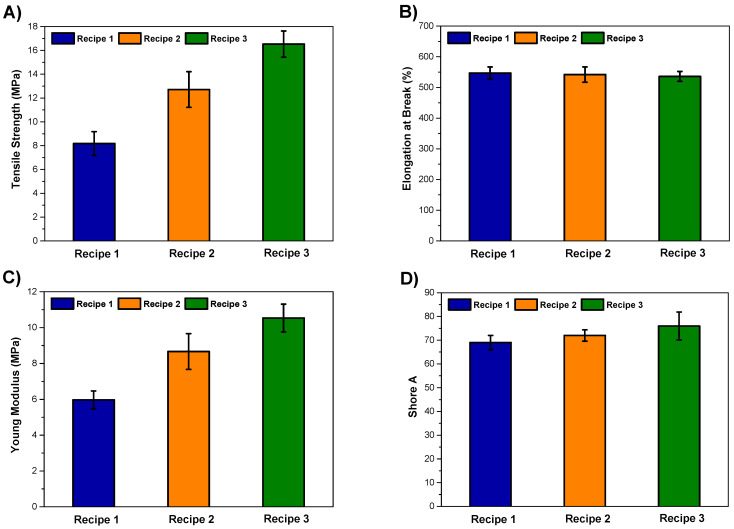
(**A**) Tensile strength, (**B**) elongation at break, (**C**) Young modulus, (**D**) Shore A values of composites.

The temperature-dependent relaxation modulus of NR/SBR/CB composites with varying MWCNT loadings at 3 and 7 phr can be seen in the graphical representation provided in Figure 6. The composite material comprising NR/SBR filled with hybrid fillers consisting of carbon nanotubes exhibited slightly higher initial modulus at lower temperatures and elevated modulus at specific temperatures when compared to NR/SBR/CB composites. As reported by Vennemann et al. [57,59,60], the marginal escalation in stress within the temperature span of up to 40 °C is primarily ascribed to the entropic elastic forces exerted by the rubber polymer chains. Above 45 °C, the stress decreases linearly with temperature before a strong stress decay that occurs at temperatures above 110 °C. The chemical coupling of rubber to the filler surface, which contrasts with the Payne effect, can also be detected when used with low filler content. The intermediate decrease in stress in the temperature range reflects physically induced stress relaxation caused by polymer filler interactions. The subsequent thermal processes occurring in the temperature range spanning 40 °C to 105 °C are predominantly linked to the phenomenon of debonding, where the interfacial connections between the rubber and the filler material undergo disruption. However, the scission of rubber chains emerges as the dominant mechanism responsible for the substantial reduction in stress observed within the temperature range surpassing 105 °C. In the context of the present study, it becomes evident that the stress within all composites diminishes notably between 45 °C and 120 °C. However, for NR/SBR composites with higher MWCNT content, it happens at an elevated stress relaxation modulus within this temperature range. Moreover, as seen from Table 5, NR/SBR compounds with 7 phr MWCNT nanoparticles exhibited the highest initial stress σ0 and T10 values, which is associated with the thermoelastic inversion point, which can be attributed to higher cross-link density, consistent with cure extent values. All these observations may be attributed to the higher amount of crosslink structures, as well as intensified filler–filler and filler–rubber interactions (as depicted in Figure 2). These combined factors contribute to the augmentation of NR/SBR/CB/MWCNT composites when contrasted with NR/SBR composites that lack the incorporation of MWCNTs. Notably, this phenomenon aligns with the discernibly greater torque difference (as outlined in Table 4), the elevated 100% modulus (as indicated in Figure 7), and the enhanced cross-link density (as reported in Table 5) [61]. Moreover, relaxation spectra (H(T)) of NR/SBR compounds, including single and hybrid filler, in the temperature range from 30 °C to 120 °C can be seen in Figure 7. As seen in Figure 7, the relaxation spectra of the NR/SBR compound with 7 phr MWCNT showed an upward trend in comparison with the other compounds. This can be attributed to the improved distribution of MWCNT in the rubber matrix, an enhanced interfacial area between MWCNT and NR/SBR resulted in improved initial relaxation modulus and increased physical desorption [62,63].

At a constant strain, the slope of the stress versus temperature plot can be used to determine the cross-link density (CLD) of a material [64,65]. As shown in Table 5, the CLD of MWCNT-reinforced tire tread NR/SBR compounds is higher than that of CB-reinforced compounds. Moreover, it increased as the MWCNT content increased, which is consistent with cure extent values. In addition, the determination of crosslink densities in the vulcanizates was also conducted through the utilization of the equilibrium swelling method. As presented in Table 5, there is a discernible upward trend observed in the cross-link density of the vulcanizates as the MWCNT content is increased. This can be primarily attributed to the establishment of physical cross-links between the CNTs and the polymer matrix, thereby immobilizing the polymer chains and impeding solvent transport, as reported by previous studies [66,67,68]. Moreover, as mentioned before, a homogeneous distribution of MWCNTs in NR/SBR/CB matrix using a high-torque lab-scale micro-compounder resulted in an improvement in the CLD of composite materials. 

### 3.4. Mechanical Properties

To evaluate the effect of hybrid filler on the mechanical properties of NR/SBR tire tread compounds, tensile, hardness, and abrasion tests were carried out. The changes in mechanical properties are shown in Figure 7 and tabulated in Table 6.

The reinforcing effect of fillers in rubber matrices is the result of several molecular mechanisms involved in this complex phenomenon: the rubber network, the hydrodynamic effect, the filler–filler interactions, and the filler–rubber interactions [19,69,70,71]. In contrast to NR/SBR/MWCNT composites, the incorporation of carbon nanotubes has yielded a striking increase in the material properties of tensile strength within the rubber matrix. At an MWCNT concentration of 3 phr, a notable enhancement of 55% in the tensile strength was observed in comparison to the NR/SBR/CB. Upon further increasing the MWCNT content to 7 phr, a remarkable upsurge of 102% was realized in the tensile strength. The absence of conspicuous MWCNT aggregates within the rubber matrix can be easily seen in the SEM images. Instead, a good distribution of MWCNT nanoparticles indicates the establishment of robust interactions between the MWCNTs and the polymer matrix. Consequently, these interactions endow the MWCNTs with the capacity to effectively transmit stress throughout the rubber matrix, thereby substantiating their pivotal role in enhancing the tensile strength of the resultant nanocomposites [24,49]. 

Figure 7C,D illustrate the variation in the Young modulus and hardness values of NR/SBR/CB at various MWCNT loading ratios. As seen, both the modulus and hardness values of composites are significantly enhanced with the incorporation of MWCNT nanoparticles. This trend underscores the notable impact of MWCNTs in augmenting the rigidity of the macromolecular chains in NR/SBR to a greater extent than carbon black [25]. Moreover, the good distribution of MWCNTs in the rubber matrix created a larger interfacial area between hybrid fillers and NR/SBR matrix that significantly improved the stiffness of the resultant composites. Additionally, in line with findings by Shanmugaraj et al. [50], nanoparticles with substantial aspect ratios contribute to the formation of supplementary entanglements and physical cross-links within the natural rubber matrix. Consequently, NR/SBR/CB/MWCNT hybrid nanocomposites exhibited enhanced stiffness. However, the elongation at break values slightly decreased with the addition of MWCNT nanoparticles to the rubber matrix, as expected. The reduction in elongation at break can be ascribed to the diminished deformability arising from the stiffened interface between MWCNTs and the rubber matrix [72]. The inclusion of fillers serves to fortify the rubber composite, thereby engendering elevated abrasion resistance and reduced volume loss. As shown in the SEM images, it is evident that the MWCNT particles exhibited homogeneous morphologies, thereby expanding the surface area available for interaction between the rubber matrix and the filler. This characteristic augmentation in the contact surface area contributes to a notable reduction in the volume loss of the NR/SBR/CB composite, thereby indicating an improvement in the abrasion resistance, as shown in Table 6 [73].

### 3.5. TGA Results

**Figure 8:**
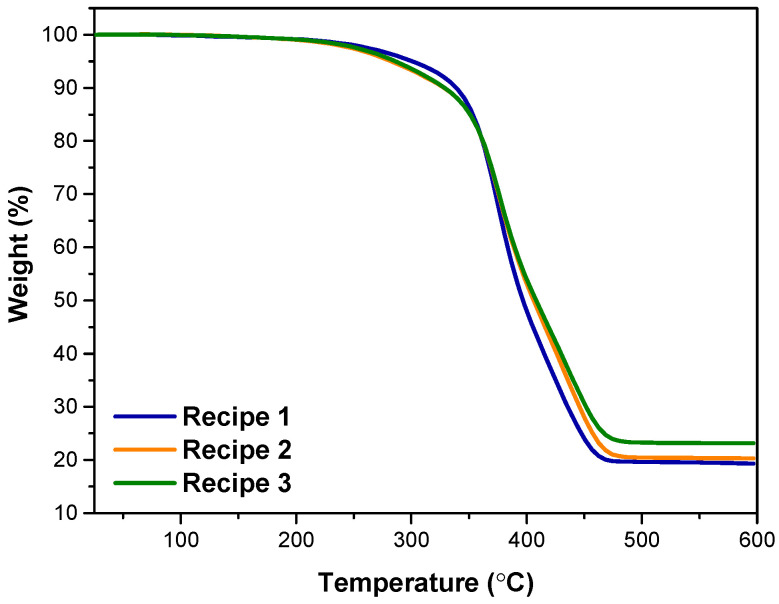
TGA curves of compounds.

The thermal stability of samples was evaluated via TGA analysis. Table 7 and Figure 8 show the thermal degradation data and TGA curves of single- and hybrid-filler-filled NR/SBR compounds, respectively. The temperatures at 5% weight loss (T_d5_), 10% weight loss (T_d10_), and maximum weight loss (T_dmax_) of NR/SBR, including reinforcements, can be seen in Table 7. Figure 8 reveals a uniform single-step degradation pattern observed across all composite materials within the temperature range of 300–450 °C under a nitrogen atmosphere. Specifically, in the NR/SBR/CB without MWCNTs, the initiations of degradation (T_d5_ and T_d10_) occurred at approximately 301.0 and 339.1 °C. However, in the presence of MWCNTs, the initial decomposition temperature started at lower temperatures, indicating the rate of degradation increased with the incorporation of MWCNTs into the NR/SBR/CB. This was attributed to the high thermal conductivity and specific surface areas of MWCNTs, which can accelerate the transfer of heat, as mentioned in the following section [48].

### 3.6. Electrical and Thermal Conductivity Test Results

The thermal conductivity test results of CB- and CB/MWCNT-filled NR/SBR composites are given in Table 8. The thermal dissipation and heat buildup (HBU) are closely related to the thermal transport properties of tire rubber. The enhanced thermal conductivity is beneficial to decrease the HBU, which, in turn, improves the service life of tire rubber [11]. As shown in Table 8, the thermal conductivity of NR/SBR/CB composite is the lowest, which is 0.56 W/mK. The thermal conductivity of MWCNT-reinforced tire tread composites is significantly increased. Among them, the thermal conductivity of the NR/SBR/CB composite, including 7 phr MWCNT, is the highest, reaching 0.91 W/mK, increased by 62.5% in comparison with the NR/SBR/CB composite. As mentioned in the literature, enhancing the distribution state of fillers holds paramount importance in the restoration of the desired thermal attributes in composites. The utilization of “polydispersity” or a “hybrid filler” approach serves as a viable method to advance the thermal properties of these composites. This approach entails the incorporation of smaller fillers at the interface, preventing agglomeration and ensuring uniform distribution alongside larger fillers. A hybrid filler system, encompassing thermally conductive fillers of varied sizes, shapes, or chemical properties, has been demonstrated to optimize distribution within the matrix and foster the creation of efficient thermal conduction pathways. For instance, in contrast to three-dimensional (3D) filler structures, one-dimensional (such as carbon nanotubes) and two-dimensional (2D) fillers with a high aspect ratio possess the capability to forge continuous, thermally conductive pathways within the polymer matrix, even when present in relatively low volume fractions [74,75,76,77,78,79,80]. Therefore, it can be concluded that improved distribution of MWCNTs in rubber matrix through high-torque micro-compounder facilitated a phenomenon known as the “dilution effect” arising from the reduction in interstitial gaps between adjacent filler particles as the filler content is increased. Consequently, the hindrance encountered by phonon-mediated thermal transport through the filler network is diminished [81].

Table 8 summarizes the electrical conductivity of NR/SBR compounds as a function of both CB and MWCNT content. Numerous critical factors, including the specific nanotube variant, the nature of the polymer matrix, the interactions between fillers and the matrix, as well as the orientation of the fillers, play pivotal roles in shaping the electrical characteristics of the material. Carbon nanotubes exhibit the capacity to confer sufficient electrical conductivity at substantially lower filler content levels, owing to their elevated aspect ratios, thus preserving the intended mechanical characteristics of the resultant material. An alternative strategy for enhancing electrical conductivity has been elucidated in studies involving the concurrent integration of hybrid fillers comprising carbon nanotubes and carbon black. It has been empirically validated that the introduction of carbon nanotubes into nanocomposites already containing carbon blacks engenders an improved overall distribution, primarily due to the synergistic interactions between these disparate fillers. This synergy results in the formation of interconnected structures, which serve to bridge the interstitial gaps between previously unconnected particles [82,83,84]. 

**Figure 9:**
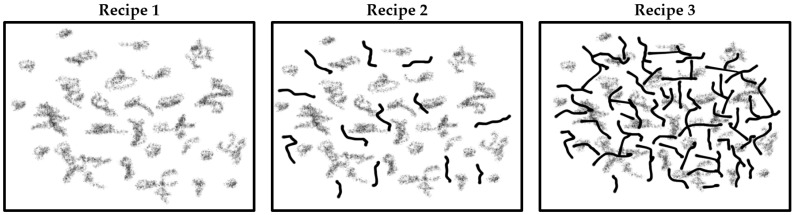
The distribution mechanism of CB and CB/MWCNT in NR/SBR matrix.

As depicted in Table 8, the addition of MWCNTs, even at low loadings, significantly increased the electrical conductivity of the NR/SBR/CB composites. A sudden decrease in the surface resistivity of samples was obtained as the MWCNT content increased to 3 phr. At a specific concentration of conductive particles, which is commonly referred to as the percolation threshold, a contiguous network of fillers is established throughout the matrix, thereby facilitating a marked transition from an insulating to a conducting state [19]. This critical threshold marks the stage at which the filler particles coalesce to form an interconnected network within the matrix. The spatial proximity of filler particles at this threshold is such that it enables the dielectric breakdown of the matrix material. Consequently, at this juncture, electrons can effectively traverse the filled rubber matrix, facilitated by mechanisms such as hopping or quantum tunneling, thereby precipitating a marked and abrupt shift in electrical resistivity [81]. As the electrical conductivity of the filler and the matrix are different by many orders of magnitude, the percolation of a filler must achieve electrical conductivity in the otherwise insulating matrix. The volume loading corresponding to a sharp increase in electrical conductivity is known as the percolation threshold. The incorporation of MWCNT as a second filler at 3 phr results in a decrease in the electrical resistivity of the NR/SBR/CB composite by an order of magnitude. The results indicated that the percolation threshold of NR/SBR was achieved at a 3 phr MWCNT loading level. In this situation, the CB aggregates were found to be uniformly distributed throughout the composite material, forming robust connections with the MWCNTs (Figure 9). This configuration established novel electron conduits within the interconnected MWCNT-CB-MWCNT networks that traverse the NR/SBR matrix [85]. In a general conclusion, as a consequence of enhanced distribution techniques, there is a discernible shift in the percolation threshold toward a lower nanotube content. Consequently, the assessments of electrical resistivity assume significance as an indirect means to appraise the quality of distribution. As highlighted in many parts of this manuscript, the state of distribution of MWCNTs plays a pivotal role in regulating performance, notably the electrical characteristics of composites reinforced with MWCNTs.

## 4. Conclusions

The intrinsic low thermal conductivity inherent in tires contributes to the phenomenon of heat build-up and concurrent material degradation. In light of this, there is a recognized necessity to enhance the thermal conductivity of tires, a goal that can be effectively realized through the incorporation of conductive fillers. Accordingly, carbon-based fillers emerge as particularly auspicious additives for augmenting the thermal characteristics of polymer composites, owing to their exceptional thermal properties. Therefore, in this study, it was aimed to decrease the heat accumulation properties as well as to improve the mechanical, rheological, and morphological properties of NR/SBR-based tire tread compounds using thermally conductive carbon nanotube nanoparticles. For this purpose, different tire thread compound recipes, including different loading levels of MWCNT, were prepared. The improved distribution of MWCNT/CB hybrid fillers was achieved by using a laboratory-scale high-torque micro-compounder, serving as an economically viable and expeditious research facility, presenting opportunities for the manipulation of small quantities of materials.

The incorporation of MWCNT nanoparticles in the NR/SBR compounds significantly developed the thermal conductivity of the samples, resulting in heat removal from the tire tread compound, which can improve the service life of a tire. Moreover, the enhancement in MWCNT distribution within the NR/SBR compound had a discernible impact on mechanical, rheological, and stress relaxation characteristics. Specifically, with an increase in the distribution of MWCNT, several key vulcanization parameters, such as scorch time and cross-link density, were notably amended. Moreover, the homogeneous distribution of MWCNT created more interfacial interaction and interfacial area between the fillers and rubber matrix; therefore, mechanical properties such as tensile strength and Young modulus were substantially developed. In addition, the findings of the study revealed that the percolation threshold for the NR/SBR composite occurred at a loading level of 3 phr of MWCNTs. It was observed that CB aggregates were uniformly distributed throughout the tire tread compound, effectively establishing robust interconnections with the MWCNTs.

## Figures and Tables

**Table 1:** Materials used in the study.

Materials	Suppliers
NR	Baria Rubber Co., Ho Chi Minh City, Vietnam
SBR	Baria Rubber Co., Ho Chi Minh City, Vietnam
CB	Elkim/N330, Istanbul, Türkiye
MWCNT	Nanografi, Ankara, Türkiye
Naphthenic Oil	Petro Yağ, Kocaeli, Türkiye
Zinc Oxide	RubberChem, Istanbul, Türkiye
Stearic Acid	RubberChem, Istanbul, Türkiye
IPPD	RubberChem, Istanbul, Türkiye
TMQ	RubberChem, Istanbul, Türkiye
MBT	RubberChem, Istanbul, Türkiye
CBS	RubberChem, Istanbul, Türkiye
PVI	RubberChem, Istanbul, Türkiye
Sulfur	RubberChem, Istanbul, Türkiye

**Table 2:** Rubber compound recipes (phr).

Components	Recipe 1	Recipe 2	Recipe 3
NR	70	70	70
SBR	30	30	30
CB	30	27	23
MWCNT	0	3	7
Oil	10	10	10
ZnO	3	3	3
Stearic Acid	2	2	2
IPPD	2	2	2
TMQ	1	1	1
MBTS	0.4	0.4	0.4
CBS	1	1	1
PVI	0.2	0.2	0.2
Sulfur	2.0	2.0	2.0

**Table 3:** DSC test results of compounds.

Compounds	T_x-link,onset_ (°C)	T_x-link,peak_ (°C)
Recipe 1	148.6	161.3
Recipe 2	151.3	164.5
Recipe 3	154.1	169.5

**Table 4:** Rheological properties of compounds.

Compounds	ML (dNm)	MH (dNm)	t_s2_ (min)	t_90_ (min)	Cure Extent (dNm)	Cure Rate Index (CRI)
Recipe 1	1.25	7.41	1.27	2.78	6.16	66.22
Recipe 2	1.43	7.88	1.34	3.00	6.45	60.24
Recipe 3	1.63	8.72	1.35	3.19	7.09	54.34

**Table 5:** TSSR results (*σ*_0_ and T10) and the cross-link density (CLD) of compounds from Flory–Rehner swelling measurements and TSSR method.

Compounds	T10 (°C) *	Initial Stress, *σ*_0_ (MPa)	TSSR (mol/m^3^)	Flory–Rehner (mol/m^3^)
Recipe 1	80.5	0.58	97.8	231.8
Recipe 2	83.0	0.40	117.9	304.3
Recipe 3	100.2	0.79	132.9	383.7

* T10 stands for the temperature at which the force (*F*) decreased by 10% from its initial value (*F*_0_).

**Table 6:** Tensile test, Shore D, and abrasion test results of compounds.

Compounds	Tensile Strength (MPa)	Elongation at Break (%)	Young Modulus (MPa)	Shore D	Abrasion Loss (%)	Abrasion Loss (mm^3^)
Recipe 1	8.2 ± 1.1	548 ± 20	6.0 ± 0.5	69 ± 3	19.8	191.1
Recipe 2	12.7 ± 1.5	543 ± 26	8.7 ± 1.5	72 ± 2	19.4	188.5
Recipe 3	16.5 ± 1.1	536 ± 16	10.5 ± 0.8	76 ± 6	10.4	131.3

**Table 7:** TGA test results of compounds.

Compounds	T_d5_ (°C)	T_d10_ (°C)	T_dmax_ (°C)	Char Yield (%)
Recipe 1	301.0	339.1	376.0	19.3
Recipe 2	282.9	328.5	377.3	20.3
Recipe 3	286.4	329.0	377.0	23.2

**Table 8:** Electrical and thermal conductivities of compounds.

Compounds	Surface Resistivity (Ohm/cm^2^)	Thermal Conductivity (W/m·K)
Recipe 1	4.5 × 10^7^	0.56
Recipe 2	6.5 × 10^6^	0.58
Recipe 3	1.4 × 10^6^	0.91

## Data Availability

The data presented in this study are available upon request from the corresponding author.
